# Large-Scale Data Analysis for Glucose Variability Outcomes with Open-Source Automated Insulin Delivery Systems

**DOI:** 10.3390/nu14091906

**Published:** 2022-05-02

**Authors:** Arsalan Shahid, Dana M. Lewis

**Affiliations:** 1 CeADAR—Ireland’s Centre for Applied AI, University College Dublin, D04 V2N9 Dublin, Ireland; 2 OpenAPS, Seattle, WA 98101, USA; Dana@OpenAPS.org

**Keywords:** glucose variability, OpenAPS, Type 1 diabetes, continuous glucose monitor, CGM, timeseries analysis, automated insulin delivery, AID

## Abstract

Open-source automated insulin delivery (AID) technologies use the latest continuous glucose monitors (CGM), insulin pumps, and algorithms to automate insulin delivery for effective diabetes management. Early community-wide adoption of open-source AID, such as OpenAPS, has motivated clinical and research communities to understand and evaluate glucose-related outcomes of such user-driven innovation. Initial OpenAPS studies include retrospective studies assessing high-level outcomes of average glucose levels and HbA1c, without in-depth analysis of glucose variability (GV). The OpenAPS Data Commons dataset, donated to by open-source AID users with insulin-requiring diabetes, is the largest freely available diabetes-related dataset with over 46,070 days’ worth of data and over 10 million CGM data points, alongside insulin dosing and algorithmic decision data. This paper first reviews the development toward the latest open-source AID and the performance of clinically approved GV metrics. We evaluate the GV outcomes using large-scale data analytics for the *n* = 122 version of the OpenAPS Data Commons. We describe the data cleaning processes, methods for measuring GV, and the results of data analysis based on individual self-reported demographics. Furthermore, we highlight the lessons learned from the GV outcomes and the analysis of a rich and complex diabetes dataset and additional research questions that emerged from this work to guide future research. This paper affirms previous studies’ findings of the efficacy of open-source AID.

## 1. Introduction

More than 536.6 million people globally (10.5% between the ages of 20–79) are estimated to be living with diabetes [1]. Regardless of type of diabetes, in the US alone, 150–250 million people are estimated to be living with insulin-requiring diabetes [2]. The tools for insulin administration have improved over the last decades, moving from syringe injections to insulin pens and insulin pumps. Glucose monitoring technology has also improved with the advent of continuous glucose monitors (CGM), which enable the measurement of interstitial glucose levels every few minutes. While interstitial glucose does have a lag time, the frequency and trend of more-frequent measurements enable more precise insulin dosing than is possible with single point-in-time fingerstick blood glucose data measurements.

Traditionally, insulin pumps and CGMs were standalone devices that did not communicate with one another, and people with diabetes (PwD) were required to assess data from both the CGM and insulin pump to determine when insulin dosing should be adjusted. As a result, while glycemic outcomes and variability improved with this technology, they still heavily required PwD to take on the burden of diabetes management and decision making hundreds of times per day.

The next evolution in diabetes technology was the shift toward closed-loop systems, where the pump and CGM data are processed through an algorithm (on the pump or on a separate mobile device) and adjustments to insulin dosing are auomatically enacted. These are now known more broadly as automated insulin delivery (AID) systems. Some of the early systems were considered to be “hybrid” closed-loop systems (HCL), because due to the capability of the system and the timing of insulin, PwD were still required to enter a meal estimate or carb entry and also initiate manual insulin dosing for meals (meal-time bolus) [3]. These AID systems have continued to evolve and advance, and some of the latest systems are approaching “fully” closed-loop systems that require less input and interaction by the human user [4].

Typically, medical device technology is created and distributed through commercial channels. However, due to the lag in innovations in diabetes technologies, some individuals living with diabetes leveraged off-the-shelf hardware connected with custom-built software and algorithms, combined with existing on-the-market insulin pumps and CGM, arriving at an automated insulin delivery system. The first of these systems is known as OpenAPS and was shared as open source many years before the first commercial AID system was available [5]. As a result, thousands of PwD worldwide have accessed open-source AID systems, and there is estimated to be dozens of millions of hours of diabetes data from these individuals [6]. Open-source AID has been repeatedly assessed as safe and effective when compared to commercial AID [7] as well as to other methods of diabetes treatment including standard insulin pumps (continuous subcutaneous insulin infusion (CSII)) [8].

The data from early AID adopters can be used to understand not only the capabilities of the systems, but to assess and analyse the undiscovered trends and phenomena in diabetes. This includes deeper assessments and gaining knowledge around glycemic variability, which after HbA1c and time in range (TIR) is becoming a recommended metric to assess clinical outcomes for PwD [9,10].

### 1.1. Automated Insulin Delivery (AID) Technologies

Automated insulin delivery systems leverage three components: an insulin pump, a CGM, and an algorithm to communicate and process the data from both devices. The algorithm can be contained within the physical body of the insulin pump or held on a separate mobile device. If the algorithm is held on a separate mobile device, decisions are communicated via Bluetooth to a connected and authorised insulin pump. Some open-source AIDs use a small radio bridge device and translate Bluetooth commands to 900 MHz radio-frequency, which older insulin pumps were programmed with.

Because of the early adoption of open-source AID, such as OpenAPS [11], there was early interest from clinical and research communities regarding data and outcomes from such systems, especially because they were self-built by patients. Early studies performed on OpenAPS were primarily retrospective studies [12,13,14] and in some cases relied on self-reported data. Studies that followed began analysing retrospective data (that were not self-reported) to further assess outcomes with open-source AID. However, a majority of studies focused primarily on high-level outcomes of average glucose levels, estimated HbA1c, and overall TIR metrics without looking deeper into system features or concrete concepts such as glycemic variability [15,16,17,18].

With ever-increasing interest in accessing data from open-source AID users, a method was formulated to allow individuals to anonymously and seamlessly donate data for research. The goal was to remove the burden on individual PwD who wanted to support research but did not want to be frequently contacted individually and asked to share their data. A key developer and founder of OpenAPS worked to build the *OpenAPS Data Commons* on the *Open Humans platform*, which enabled individuals to anonymously upload their data and share it [19]. The OpenAPS Data Commons then coordinated with interested researchers to manage access to the anonymised pool of data. All individuals have insulin-requiring diabetes by virtue of using an open-source AID. The dataset includes individuals using a variety of open-source AID, such as *OpenAPS* (OpenAPS, https://OpenAPS.org/, accessed on 25 April 2022), *AndroidAPS* (AndroidAPS, https://androidaps.readthedocs.io/en/latest/, accessed on 25 April 2022), and *Loop* (Loop, https://loopkit.github.io/loopdocs/, accessed on 25 April 2022).

The OpenAPS Data Commons is a rich dataset that includes CGM data with timestamps, insulin pump dosing data, and a log of algorithmic processing such as glucose predictions and dosing decisions made by the open-source AID. Other features include any manual entries by PwD such as carbohydrates or temporary targets. A previous version of the OpenAPS Data Commons (when *n* = 119) was assessed to have 46,586 total days’ worth of data and over 10 million CGM data points [20].

Based on CGM glucose data alone, this is one of the largest glucose datasets available from individuals with insulin-requiring diabetes. Most studies with “big data” approaches on diabetes technologies perform analysis on limited-scale real-world datasets. Some examples include employing random forest and support vector machine algorithms using 14 days’ worth of data collected from 25 people [21], while others include testing in 10 people across 4 weeks to achieve 255 days of data after training on 27,466 days from an unspecified number of individuals [22]. The next largest dataset used in diabetes research appears to be from the Tidepool Big Data Donation Project with up to 41,318 days of data from *n* = 175 individuals as used in at least one paper [23]. The Tidepool Big Data Donation Dataset may have more data available [24], although it requires a fee to license the datasets for research [25], which may be one reason a limited portion of the dataset is more frequently used. The OpenAPS Data Commons, at the time of writing this paper, includes more than 184 individuals’ donated data and surpasses the size of the number of days of data from other datasets reported in the literature. This paper uses the *n* = 122 version that was the size of the dataset at the start of this paper’s analysis work; additional data were donated to the dataset following the commencement of this paper.

In addition to the increased number of days, total number of individuals, and overall glucose and insulin dosing dataset, the potential from the OpenAPS Data Commons dataset also comes from the additional data collected from the AID with both a log of all insulin dosing performed by the system and human-entered inputs (such as carbohydrates consumed, targets adjusted for exercise, etc.) as well as the recording of algorithmic processing by the open-source AID system.

As a result of the OpenAPS Data Commons, it is now possible to assess research questions and expand the knowledge of insulin-requiring diabetes in ways that were not possible before due to a lack of truly large-scale diabetes data.

### 1.2. Motivation to Study Glucose Variability

There has been increased interest in glycemic variability, both as a potential metric to assess outcomes in PwD as well as a metric that could be used to assess and evaluate changes in other metrics such as correlations with hypoglycemia [26]. Previous studies have found glucose variability to be a predictor of severe hypoglycemia [27], but the exact relationship between them is unknown. According to the Diabetes Complications and Control Trial (DCCT), one of the concerns in insulin-requiring diabetes is that lower average glucose and HbA1c might be achieved by increasing rates of hypoglycemia and resulting correlations with increased morbidity. Modern diabetes technology typically enables lowered average glucose and HbA1c without increasing rates of hypoglycemia, yet the risk of hypoglycemia still exists, even with AID. Furthermore, hyperglycemia and glycemic variability are considered to be a factor inducing oxidative stress and overproducing reactive oxygen species. With AID data, such as OpenAPS Data Commons, it is possible to assess rates and incidents of hypoglycemia and hyperglycemia and study their exact relationship with glycemic variabilty.

It is also possible to assess whether there are any differences within subgroups in the dataset, such as by self-reported gender identity, age, or other basic demographic variables. Currently, all PwD using AID use the same algorithms and adjust their baseline settings individually. However, if any sub-group can be identified with different glucose outcomes from using the same system, it may be possible to either adjust systems in the future or recommend individuals to change settings or feature choices to better address differences within groups using the same AID.

### 1.3. Paper Contributions and Organisation

This paper seeks to describe methods of assessing glycemic variability using the CGM data from the largest freely available dataset of individuals with insulin-requiring diabetes, the OpenAPS Data Commons, along with a partial self-reported dataset of basic demographic variables.

In summary, the main original contributions of this work are:

- Methods and techniques for data cleaning and glycemic variability analysis for large-scale diabetes data, i.e., OpenAPS Data Commons, originating from open-source AID technologies. We further calculate standard clinical metrics to analyse the glucose variability outcomes and add to previous demonstrations of the effectiveness of open-source AID technologies.
- The application of a machine-learning-based hierarchical clustering algorithm in order to understand distinct patterns across glucose profiles of insulin-requiring individuals. We discovered that there are no obvious sub-populations in the open-source AID user community that are being underserved.
- The first in-depth timeseries analysis for glucose variability outcomes and data-driven comparative analysis of the outcomes in open-source AID users based on self-reported population genders. We demonstrate some gender-wise differences in glucose mean and distribution during the times of a day, days of a week and month, and months of a year.

All programming scripts and tools developed for the analysis of demographics and glucose data in this paper are made public at [28].

Section 2 presents the literature review of existing glucose analysis software tools and technologies making use of CGM data and provides a comprehensive analysis of state-of-the-art research and challenges in glycemic variability. In Section 3, we provide methods and techniques adopted for diabetes data collection, anonymisation, and cleaning along with a list of employed glucose analysis metrics in this paper. Section 4 presents the results of demographics and glucose data analysis conducted in this paper, including the timeseries analysis of glycemic variability outcomes in the OpenAPS Data Commons dataset. Section 5 presents discussions on the analysed glucose outcomes, highlights the lessons learned and criticises the limitations, and provides a roadmap for future considerations. Section 6 concludes the paper.

## 2. Related Work

This section reviews the existing tools and techniques in the area of glucose variability analysis for continuous glucose monitoring (CGM) data and highlights the major challenges.

### 2.1. Popular Metrics for Assessing Glucose Variability

Insulin-requiring diabetes management is commonly aided by CGM, which yields longitudinal glucose data and assists clinicians and researchers to understand various factors of glucose variability. Since CGM generates timeseries data, there are a number of statistical and machine-learning (ML) methods to help understand and summarise it. According to PubMed, the glucose variability keyword is mentioned in over 26,000 publications and is considered a key metric in clinical research [29]. There are over 25 glucose variability (GV) metrics proposed in the literature, and only a few have been clinically validated. Since the reported results and statistics do not converge based on a single methodology, comparing and analysing them across various studies is a non-trivial task. Table 1 lists the clinically validated metrics that employ CGM data to calculate glucose variability.

### 2.2. Software Tools for Automated Variability Analysis of Continuous Glucose Monitoring (CGM) Data

Cgmquantify [37] is an open-source toolbox developed to assist the calculation and visualisation of various clinically validated metrics of glucose variability. Its functions are implemented as Python and R programming libraries but require glucose data in Dexcom CGM format. In [39], the CGM-GUIDE tool is proposed that provides a user-friendly graphical user interface for monitoring CGM data and different variability metrics including the percentage of time spent in different glucose levels during an interval. The CGDA [40] data analysis tool implemented as an R programming package provides a simple interface and list of functions to analyse glucose data from any available CGM.

Other software tools featuring support for glucose management and variability analysis include EasyGV [41], cgmanalysis [42], and GlyCulator [43].

### 2.3. Comprehensive Review of Efficiency and Performance of Glucose Variability Metrics

Although the DCCT had considered HbA1C as the standard metric for glycemic control (in part due to the different set of tools available at the time of the landmark study), minimising the blood glucose variability (GV) has been more recently explored over the past decade as another promising metric for glucose management [44]. Understanding GV is integral to both the physiology and pathophysiology of diabetes, and it is interlinked with the risk of hypoglycemia. Quantifying the interconnection between GV and hypoglycemia is tough as the glucose fluctuations are determined by both the amplitude and timing [45].

Simple statistical metrics such as mean blood glucose and standard deviations have not been observed to be contributing factors in the development of microvascular complications of diabetes [46]. A strong correlation has been shown between HbA1c and diabetes-related complications for both Type 1 and Type 2 diabetes [47]. However, in recent years, instability of glucose has been found to contribute more than HbA1c in the development of diabetes-related complications. This has been further validated by clinical studies where significant glucose fluctuations have been observed in children with Type 1 diabetes while they maintain a normal HbA1c. A review of medical conditions such as oxidative stress and intensive care settings shows a difference in GV [47].

It has been discovered that GV increases progressively from initial diabetes-related conditions through the development of Type 2 diabetes and is comparatively higher in people with Type 1 diabetes. A review of GV metrics in clinical and research applications shows that the coefficient of variation (CV) and standard deviation (SD) are the most used metrics for GV in the literature [9].

A critical analysis of the efficiency of the mean amplitude of glycemic excursion (MAGE) shows that it lacks the ability to capture excursion frequency and distance travelled. Another drawback of using MAGE as a GV metric is the differences in its implementations [48]. The available MAGE implementations are provided by EasyGV, cgmanalysis, cgmquantify, and iglu, yielding median errors of 20%, 78%, 11%, and 42%, respectively, when compared with the manual calculations. In Fernandes et al. [49], an approximation algorithm to access MAGE is developed with an objective to improve the accuracy of calculations. The accuracy of the technique was evaluated using a five-fold cross-validation technique and was found to have a median error of 1%.

In Marling et al. [50], CGM charts were classified using three ML models including a naive Bayes classifier, a multilayer perceptron, and a logistic model tree. For model training, daily CGM curves were labelled by two physicians and compared to GlycoMark [51] (serum levels of 1,5-anhydroglucitol) as reference measures. However, the labelling consistency between the two physicians was around 81%. Furthermore, the performance of ML models was also affected by the limited training data.

Dovc et al. [52] evaluated the correlation between CGM use and GV in pre-school children with Type 1 diabetes using data from the Slovenian National Registry. GV was analysed for a period of 5 years with and without the use of CGM among the participants. The results indicate that the use of CGM reduced the GV. The mean glucose with and without CGM was 3.6 mmol/L and 4.3 mmol/L, respectively. Similarly, the coefficient of variation was 44% and 46.1% with and without CGM use, respectively.

In Moscardo et al. [41], the glycemic variability assessment methods are developed in the EasyGV software tool that yields a correlation of 98% with most of the GV metric except the calculation of MAGE. The difference in calculating MAGE is because EasyGV calculates MAGE using a fuzzy-logic-based method.

Boris et al. [26] argued that diabetes management requires balancing between mean blood glucose and frequency of hypoglycemia. Therefore, with the adoption and evolution of the automated insulin delivery systems (AID), it is necessary to standardise the GV metrics. The authors computed various metrics including SD, CV, MAGE, MAG, LBGI, HBGI, and ADRR to understand the principal components of glucose variations, i.e., amplitude and timing.

The existing GV metrics mainly focus on measuring the amplitude components of the fluctuations and lack an integration of the timing component [45]. However, because of the inherent inaccuracies of various existing GV metrics, such as J-index, MAG, and CONGA, there is a need to develop and fine-tune metrics that characterise primary features of glucose activity such as time to peak, and time to recovery to the baseline [53]. With the evolution of CGM-based data collection technologies, calculating timing factors has become possible. Timeseries analysis techniques serve as promising techniques to measure the timing components of the glucose fluctuation profiles. The metrics for GV calculations include time in range (TIR) and time out of range (TOR).

In Rodbard et al. [9], a methodology using timeseries analysis to profile CGM data during the day is proposed to understand the relationships of GV during and after meal intakes by setting specific time windows. The need for large-scale diabetes datasets is highlighted for different populations to interpret GV and other CGM data metrics. This would further serve to better understand the practical pros and cons of traditional timeseries approaches to analysing glucose data.

Although experimental discoveries suggest oxidative stress has a strong link with short-term GV, the mechanisms for long-term GV based on visit-to-visit measurements of HbA1c and fasting plasma glucose along with their SD and CV calculations are not well defined [10].

Siegelaar et al. [54] reviewed the research methods to measure GV with a view to finding a link between GV and oxidative stress and extreme out-of-whack glycemic conditions. GV has been found to be an important predictor variable for extreme hypoglycemic conditions in people with Type 1 diabetes. GV is greater in people with diabetes who experience severe hypoglycemia [27]. However, the exact relationship is still unknown. In order to find the predictive power of the model variables for hypoglycemia, a general boosted model was developed, and coefficient of variation and MAG were found to have over 40% and 10% influence, respectively [27].

To summarise, understanding glucose variability is promising for improving diabetes management. Major challenges in the area of GV include a lack of consensus on the best approach to measure the GV given the CGM data. A better understanding of GV metrics fed by the evidence from the evaluation of real-world diabetes datasets can result in better tools to minimise the metabolic ups and downs of blood glucose levels and prevention of or reduction in, wherever possible, diabetes-related complications.

## 3. Materials and Methods

This section first details the procedures and setups put in place for diabetes data collection and anonymisation. We then describe the methods used to establish the data cleaning pipeline and list the statistical and variability metrics for glucose analysis used in this paper. Lastly, the section provides a summary of the cleaned OpenAPS Data Commons dataset and the self-reported demographics of the insulin-requiring AID users.

### 3.1. Diabetes Data Collection and Anonymisation Highlights

The primary dataset for this analysis comes from the OpenAPS Data Commons, a dataset collated as a project on the Open Humans platform. The Open Humans platform (Open Humans platform, https://www.openhumans.org/, accessed on 25 April 2022) allows individuals to connect their data to the platform and donate it to projects such as the OpenAPS Data Commons (OpenAPS Data Commons project on Open Humans, https://www.openhumans.org/activity/openaps-data-commons/, accessed on 25 April 2022). They are typically donated through an uploader project to Open Humans that anonymises the data [55], so that data stored in the Open Humans platform and then donated to the OpenAPS Data Commons are anonymised. Additionally, the OpenAPS Data Commons leverages other privacy-preserving features of Open Humans, including no collection of username or email addresses. Project participants are assigned a random, 8-digit identifier for the project. Participants can be contacted only via the Open Humans messaging platform, which again does not provide any identification of participants to the project administrator. As such, the OpenAPS Data Commons holds a complex, rich anonymous dataset that can then be used by research projects such as the one described within this paper.

The OpenAPS Data Commons dataset is provided upon request to researchers who agree to simple terms of use of the data. Once agreed upon, researchers receive a Dropbox link holding two versions of the dataset. One is an untouched, .gzip version of the dataset downloaded from OpenHumans. The second is an unzipped version that has also been converted to csv format, using an open-source tool designed to convert complex data files without known data structures [56]. The unzipped json version files also are provided.

Part of the reason for the complexity and unspecified file structure for each participant within the OpenAPS Data Commons dataset is due to the nature of the flexibility of the diabetes open-source community and the tools commonly used, such as Nightscout. Nightscout is an open-source remote monitoring platform that many use for real-time personal monitoring of diabetes data from multiple devices; other features include data analytics. Each Nightscout site is self-managed, so individuals coordinate their own data storage. Nightscout has typical device fields and data structures for common devices such as continuous glucose monitors (CGM), insulin pumps, blood glucose meters, etc. However, because a plethora of devices can be connected to Nightscout, there is often disparate formatting with regard to data labels or date and timestamps across devices. Such complexity is also therefore transferred into the data structures within the OpenAPS Data Commons, since Nightscout is typically the platform most commonly used for data donation. As a result, significant data cleaning must be done to provide uniformity across individuals’ datasets before further analysis.

#### Demographics Data Collection

Alongside the diabetes data discussed above, there is also a partial dataset of demographics information associated with the OpenAPS Data Commons. At the time that individuals first join the OpenAPS Data Commons project on Open Humans, they are redirected to a Google Form which asks for voluntary demographics information. This includes age, gender, geographic location, ethnicity; estimated weight, height, and insulin usage; relevant diabetes dates (diagnosis, pump or CGM commencement, open-source AID commencement); and type of open-source AID.

Participants are not required to fill out this survey, so some either do not see the redirect or choose not to fill it out. Gender was added as a question to the survey after the project was established, so early participants do not have gender-reported data. As the data are self-reported and only reported at the initial joining of the project, they reflect demographic data such as estimated height, age, weight, etc., only representing that point in time, and may not be representative throughout their data, as many individuals have donated multiple years’ worth of diabetes data.

Like the OpenAPS Data Commons dataset, the companion demographics file is made available via a Dropbox link for download as a .csv or .xls file upon request.

### 3.2. Glucose and Demographics Data Cleaning

The data pre-processing and cleaning pipeline for glucose and demographics data was implemented using Python frameworks and shell scripts.

**Figure A1:**
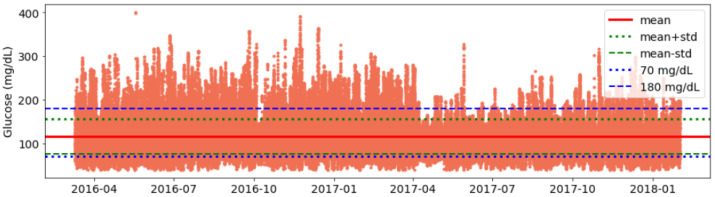
Visualisation for Glucose Data for an Open-Source AID User.

1. File formatting: We use glucose entries files in .csv format to ease data visualisation support using spreadsheets. Since the originating source contains a large volume of .json data, there are multiple .csv files for each individual. The files were previously converted to .csv using the Unzip-Zip-CSVify-OpenHumans-data.sh script (this is an open-source script along with other open-source tools used for processing large complex data such as the OpenAPS Data Commons data coming from the Open Humans platform, https://github.com/danamlewis/OpenHumansDataTools, accessed on 25 April 2022).
2. Unified datastore: We pull glucose entries data in .csv format for all individuals to a common directory representing a unified data store.
3. Timestamp cleaning and consistency: Each glucose entry file contains inconsistent timestamps. The cleanest timestamps were appended with letters such as T and Z represented in the following example format: “2018-08-04T23:58:50Z”. These were cleaned simply by trimming the alphabet letter. Multiple types of timestamp formats represented in different time zones—GMT, PDT, CES, CST, etc.—were found in single glucose entries files. We cleaned such instances programmatically by employing “regex” functions exposed by the Python Pandas package and lambda functions. Furthermore, we noticed an overlap of different formats of timestamps between the data rows. Some timestamp values were accompanied by the abbreviations for the day of the week such as “Mon”, “Tue”, “Wed”, etc., alongside the year and date. Although a lot of the inconsistent timestamps were cleaned programmatically, some of them required manual labour efforts as well. After all the pre-processing, we converted the timestamps to a consistent date–time format.
4. Glucose entries cleaning and consistency: To maintain the consistency of glucose entries, we performed the following steps during the data cleaning phase:
  - We noticed some glucose data samples contain text such as “null”. Data rows with “null” were removed in each glucose entry file.
  - Multiple .csv files for the same individual were merged, and data were organised in the increasing order of the timestamps in dataframe columns.
  - All the duplicate timeseries values (if any) were removed, and only the first entry for duplicate timestamps was kept.
  - All infinite numbers represented as “inf” were replaced with “NaN”, and all rows with “NaN” were dropped.
  - Based on CGM device knowledge, decisions were made to remove data values that represent error terms, as they should not be included in calculations for glucose values (units mg/dL). This includes removing every data point less than 39 and greater than 1000. Any data point greater than 400 and less than 1000 was replaced with 400. No further interpolations were performed to cover the error terms.
5. Validation of data consistency: Finally, we plot glucose data and visualise it for each individual to identify any anomalies or inconsistencies in timestamps and corrected them (See Figure A1).

Since demographics data collection was a self-reported process, there was a difference in the format and units of the reported data. Due to the range of errors in reported demographics, a manual data cleaning process was employed to develop a consistent and reliable dataset for use in this paper.

### 3.3. Glucose Analysis Metrics

The following statistical and variability metrics for glucose analysis are calculated in this paper.

- Count—Total CGM points to calculate the amount of data.
- Mean—Average of CGM data.
- Min—Minimum CGM data value.
- Max—Maximum CGM data value.
- Q1 or 25%—First quartile.
- Q2 or 50%—Second quartile.
- Q3 or 75%—Third quartile.
- IQR—Interquartile range.
- SD—Interday standard deviation of CGM data.
- CV—Interday coefficient of variation.
- TOR < 70—Time outside range, i.e., hypoglycemia. Total glucose data points less than 70 mg/dL in percentage.
- TIR—Time inside range, i.e., total glucose data points within target range between 70 mg/dL and 180 mg/dL in percentage.
- TOR > 180—Time outside range, i.e., hyperglycemia. Total glucose data points greater than 180 mg/dL in percentage.
- POR—Total percentage of time outside range (range in standard deviations from mean).
- J_index—Glycemic variability.
- LBGI—Glycemic variability metric to calculate low blood glucose index.
- HBGI—Glycemic variability metric to calculate high blood glucose index.
- GMI—Glycemic management indicator.

### 3.4. Summary of Glucose and Demographics Data

**Figure 1:**
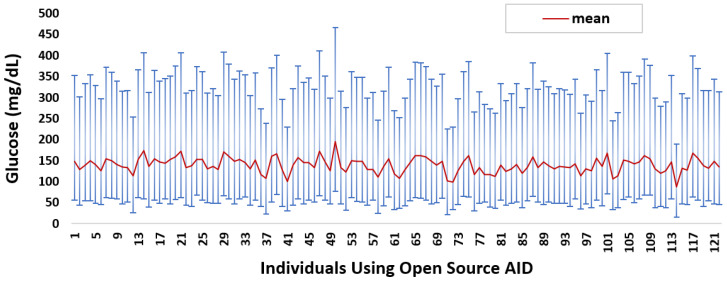
Glucose mean and distribution for insulin-requiring individuals using open-source AID systems. Total number of individuals (*n*) = 122. Average glucose mean and SD across the individuals is 139 ± 49.8.

In total, we cleaned 46,070 days’ worth of glucose data donated by 122 individuals. On average, each individual donated 377 days’ worth of data. Over 70% of the insulin-requiring individuals donated data representing five months or more. Figure 1 summarises the mean glucose and SD ranges for each insulin-requiring individual. The average mean and distribution of glucose data is 139 ± 49.8 mg/dL, respectively. The mean glucose data quartiles Q1, Q2, Q3, and interquartile range (IQR) are {102.83, 129.40, 166.13, 63.3} mg/dL, respectively.

Table 2 lists the collected demographic features alongside the count of reported demographic features. The average reported age at the point in time in which individuals donated data is 36 years. A total of 78 individuals reported their gender, out of which 50 are males and 28 are females. Insulin-requiring individuals from 21 countries reported their demographics. Most are from the USA, Germany, and UK with a total count of 45, 12, and 6, respectively. The average and median per-day self-reported insulin intake, in general, is 44.57 and 40 units, respectively. For 25% of people, insulin intake is less than 31.53 units per day and more than 51.29 units per day. The average and median insulin intake reported for males is {45.21, 39.84} units and for females is {49.06, 36.85} units, respectively.

## 4. Results

This section present the results of demographics and glucose data analysis followed by glucose variability and timeseries analysis for OpenAPS Data Commons datasets based on gender classification.

### 4.1. Demographics Data Analysis

**Figure 2:**
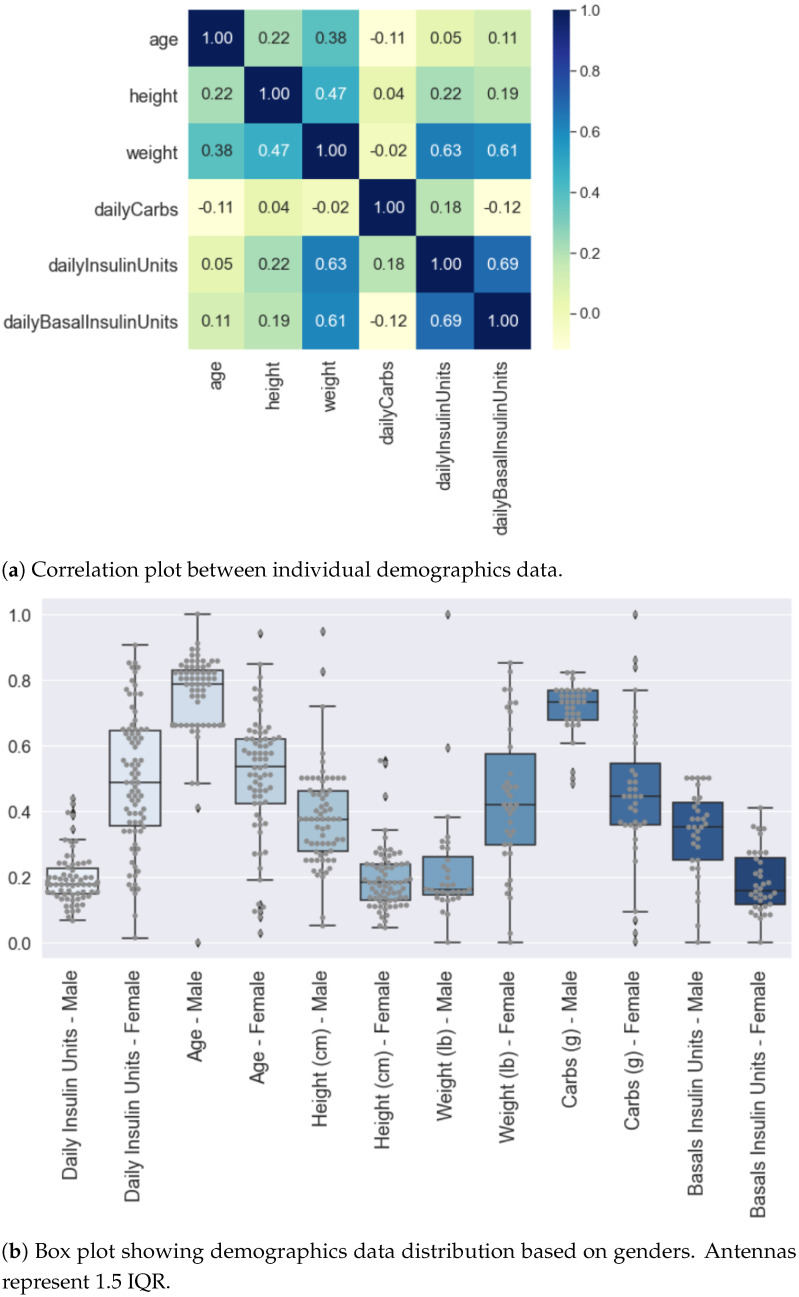
Analysis of Self-reported Demographic Attributes of Insulin-Requiring Individuals using AID Technologies. Overall data (male and female combined) are normalised using *MinMaxScaler* function provided by Python *scikit-learn* library. (**a**) Shows the highest correlation of 69% between weight and daily insulin units. Second-highest correlation is between weight and basal units equal to 61%. (**b**) The distribution of self-reported demographics with respect to gender shows a greater intake of carbs and basal in males as compared to females. Average insulin intake by males and females is 45.58 and 49 units, respectively.

To understand the relationship between the demographic features listed in Table 2, we performed statistical tests using Spearman correlation. Figure 2a shows the heat-map with correlation matrix between demographics features where light colour or 0 means no correlation and dark colour or ±1 means high (anti) correlation.

We observed a maximum correlation of 69% between self-reported total daily insulin units and daily basal insulin units. It is a statistically significant linear correlation with *p* < 0.001 within a 95% confidence interval. An increase in daily insulin by 1 unit increases daily basal insulin on average by 1.40 units. The second most statistically significant linearly correlated features are weight and self-reported total daily insulin units, i.e., 63% (*p* < 0.001). Finally, the correlation between weight and self-reported basal units is 61% (*p* < 0.001). Other demographic features show a poor and statistically insignificant correlation between each other.

Feature distributions were further analysed by classifying the data based on gender. Figure 2b shows the box plots with demographic distributions where the data are normalised altogether before classifying with respect to gender. The normalisation is performed using *MinMaxScaler* function exposed by Python *scikit-learn* Library. It can be seen that the self-reported total daily insulin intake for females is comparatively greater than males. The high positive and statistically significant correlation (*p* < 0.001) between self-reported insulin intake and weight can be observed as female weight and insulin intake are greater than the male weight and insulin intake, respectively. The average self-reported insulin intake by males and females is 45.58 units and 49 units, respectively. We observe greater carbs and basal intake for males as compared to females.

### 4.2. Glucose Data Analysis

**Figure 3:**
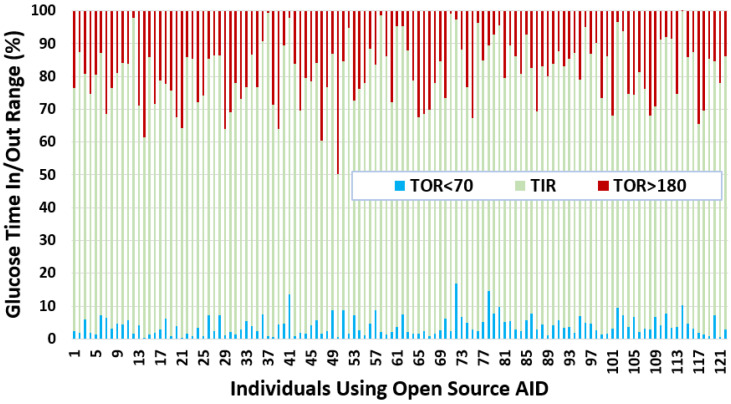
Glucose TIR and TOR for insulin-requiring individuals using open-source AID systems. Total number of individuals (*n*) = 122. The average TIR for insulin-requiring individuals in OpenAPS Data Commons dataset is 77%. There is a higher tendency for individuals to have more TOR situations over the higher TIR limit (180 mg/dL) as compared to TOR situations less than the lower TIR limit (70 mg/dL).

**Figure 4:**
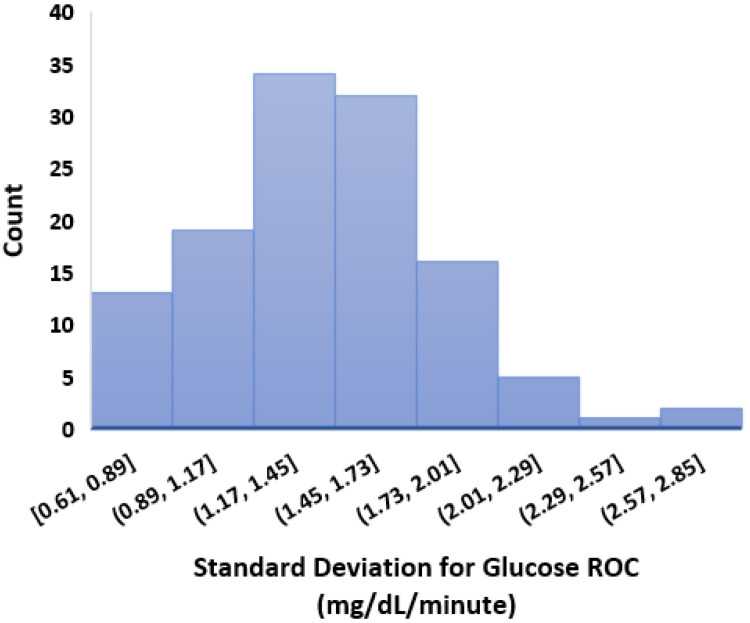
Standard deviation of glucose rate of change. Total number of individuals (*n*) = 122.

**Figure A2:**
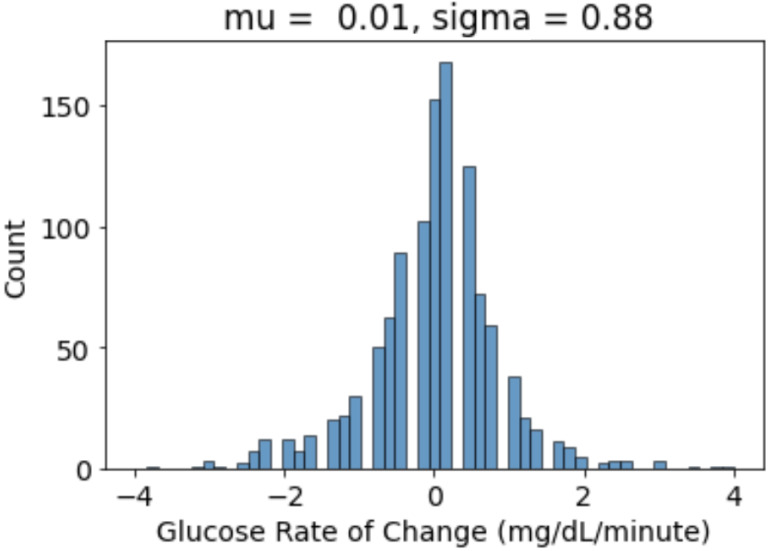
Visualisation for Glucose Rate of Change for an AID User. ‘Mu’ is mean and ‘Sigma’ is the standard deviation of distribution.

After independently profiling the glucose data for each individual (Figure A1), this section calculates the glucose analysis metrics listed in Section 3.3. Table 3 shows the summarised statistics with minimum, maximum, average, quartiles, and interquartile ranges for each glucose variability metric. Figure 3 shows a plot with portions of glucose TIR and TOR ranges in percentages for each individual in our dataset. In general, it can be seen that a number of individuals have well achieved a TIR above recommended standards [57]. If we compare the TOR < 70 and TOR > 180, we observe that there is more tendency within this dataset for an individual to have TOR > 180 than TOR < 70. Salient results include:

- It can be seen that the interday glucose standard deviation is 49.75 mg/dL with a minimum and maximum of 14.71 mg/dL and 77.33 mg/dL, respectively.
- We calculated the glucose rate of change for each individual in our dataset using the formula $\left[glucose\left(t_{i}\right)-glucose\left(t_{i-1}\right)\right]/\left(t_{i}-t_{i-1}\right)$. We analysed visually and statistically the glucose rate of change (ROC) for each insulin-requiring individual (see Figure 4). The standard deviation of glucose ROC has a minimum, average, and maximum of {0.61, 1.42, 2.69} mg/dL per minute, respectively. The Shapiro–Wilk test (statistical test for normality) performed on SD of glucose ROC data yielded *p* = 0.205 (i.e., >0.05) indicating that the data follow a normal distribution. According to 3σ rule of distribution statistics, 99.7% of the data lies between 0.29 and 2.46.
- We further analysed the distribution of standard deviation of rate of change (ROC) for individual glucose profiles in the dataset (see Figure A2). The minimum, average, and maximum of SD ROC calculated separately for males and females is {0.60, 1.36, 2.6} and {0.7, 1.4, 2} mg/dL per minute, respectively.
- The average interday coefficient of variation (CV) is 35.43. A total of 25% of the insulin-requiring individuals have CV less than 32.42 and greater than 38.47, whereas the interquartile range is 6.
- We observe that the average TIR is 77.27% for people using DIY technologies. Furthermore, less than 25% of the individuals have TIR less than 71%. However, over 25% of the insulin-requiring individuals achieved a TIR higher than 84%. The minimum, average, and maximum for TOR < 70 and TOR > 180 is {0.23%, 4%, 16.97%} and {0.05%, 18.74%, 49.67%}, respectively.
- The minimum, average, and maximum for TOR < 70 and TOR > 180 highly correlate to LBGI and HBGI, respectively.
- The mean J_index and GMI for insulin-requiring individuals in our dataset is 36.42 and 6.63, respectively.

**Table 3:** Summarised statistics for glucose variability. Total number of individuals (*n*) = 122.

	Min	Max	Average	Q1	Q2	Q3	IQR
Interday SD (mg/dL)	14.71	77.33	49.75	43.13	49.34	58.10	14.97
Glucose ROC SD	0.61	2.69	1.42	1.15	1.41	1.66	0.51
Interday CV (%)	16.86	44.94	35.43	32.42	35.87	38.47	6.05
TIR (%)	49.75	98.45	77.26	71.18	77.91	84.07	12.89
TOR < 70 (%)	0.23	16.97	4.01	1.79	3.22	5.61	3.83
TOR > 180 (%)	0.05	49.67	18.74	12.72	17.14	25.52	12.81
LGBI	0.13	3.82	1.09	0.64	0.95	1.42	0.78
HBGI	0.03	13.25	4.36	2.83	3.95	5.89	3.06
J_index	10.39	73.93	36.42	29.71	35.49	43.84	14.14
GMI	5.40	7.96	6.63	6.38	6.63	6.92	0.53

### 4.3. Clustering Glucose Profiles

To understand whether there are distinct patterns of glucose data profiles in insulin-requiring individuals in our dataset, we used an unsupervised learning approach and employed a hierarchical/agglomerative clustering algorithm.

The main steps involved in agglomerative clustering include starting by treating each glucose data profile as one cluster. Therefore, the number of clusters initially are equal to the number of glucose profiles. The clusters are then formed based on joining the closest profiles and ultimately form one big cluster by joining small clusters. Dendrograms divide the single big cluster into multiple clusters using the Euclidean distance as a metric. We used *linkage* and *fcluster* tools from *Python Scipy* library to perform the clustering.

As an outcome of our initial experimental investigation, we did not find any distinct clusters of glucose profiles. There is a possibility to obtain some distinct and meaningful clusters by tuning the distance metrics in other ML-based clustering approaches. However, we can argue that not observing distinct clusters of glucose profiles indicates that there is no obvious flaw in open-source AID systems at the highest level, which directly rebuts potential concerns related to the potential additive harm of open-source AID [58].

### 4.4. Glucose Variability Analysis Based on Gender

**Figure 5:**
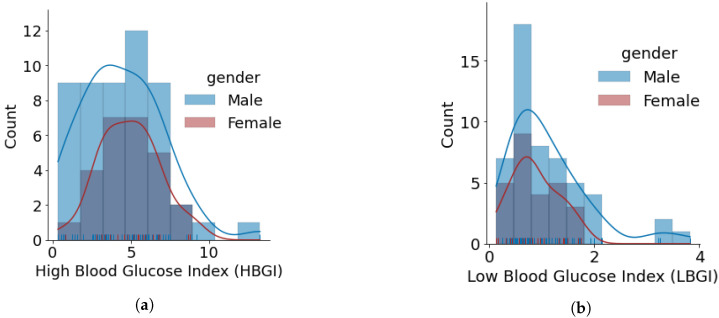

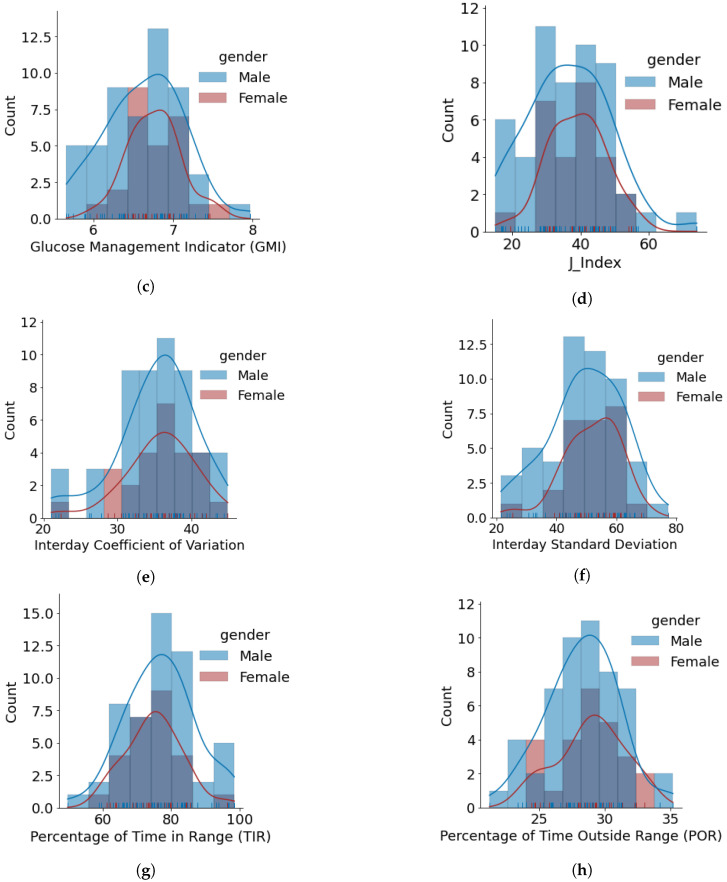
Glucose variability outcomes for individuals using open-source AID systems, based on gender. Total number of males and females is 50 and 28, respectively. (**a**) HBGI. (**b**) LBGI. (**c**) GMI. (**d**) J_index. (**e**) Glucose Coefficient of Variation. (**f**) Glucose Standard Deviation. (**g**) Glucose Percentage of Time Inside Range (TIR). (**h**) Glucose Percentage of Time Outside Range (POR).

This section presents our results for glucose variability analysis by mapping glucose entries in OpenAPS Data Commons dataset with individuals’ self-reported gender. We divided the datasets for males (*n* = 50) and females (*n* = 28) and calculated the GV metrics including HBGI, LBGI, GMI, J_index, CV, SD, TIR, and POR. Figure 5 shows the distribution of GV outcomes for insulin-requiring individuals based on gender. Salient observations from the GV outcomes include:

- The minimum, average, and maximum LBGI for females and males are {0.13, 0.87, 1.74} and {0.24, 1.11, 3.82}, respectively. Similarly, the minimum, average, and maximum HBGI for females and males is {0.80, 4.90, 8.75} and {0.33, 4.44, 13.24}, respectively. It can be seen that LBGI and HBGI distributions for males and females are positively skewed. The Shapiro–Wilk test on LBGI and HBGI yielded *p* < 0.05, confirming that the data do not belong to a normal distribution.
- The minimum, average, and maximum GMI for females and males is {6.04, 6.76, 7.46} and {5.66, 6.64, 7.96}, respectively. We do not observe any differences in GMI distribution patterns for males and females. However, the Shapiro–Wilk test yielded *p* > 0.05, indicating that the GMI follow a normal distribution.
- J_index distributions show a slight positive skew with higher average statistics for females with 38.99 as compared to males with 36.63. *p* > 0.05 is obtained from the Shapiro–Wilk test, indicating that J_index follows a normal distribution.
- There are no distinct differences between females and males for both CV and SD. The average CV in percentage and SD in mg/dL are {35.80, 51.93} for females and {35.23, 49.62} for males. Furthermore, there is a negative skew for both males and females in CV distributions. Using the Shapiro–Wilk test, *p* < 0.05 for CV confirms its non-uniform distribution. The SD distributions are more uniform (*p* > 0.05).
- Average TIR for females and males is 75.13 and 76.90. The distributions for both females and males are uniform (*p* > 0.05) and exhibit similar patterns. For our datasets, more than 75% of males and females achieve TIR of over 70%. The maximum TIR achieved for females and males in 96.25% and 98.45%
- The POR distributions for both genders are uniform (*p* > 0.05), where average POR for females is 28.92% and males is 28.26%.

#### Timeseries Analysis for Glucose Data based on Gender

This section presents the experimental results of timeseries analysis to study the variations in glucose mean and standard deviation (SD) during the hours of a day, days of week and month, and months of the year for all the individuals in the OpenAPS Data Commons dataset. We further analyse the average trends and differences between males and females in terms of glucose mean and SD.

**Figure A3:**
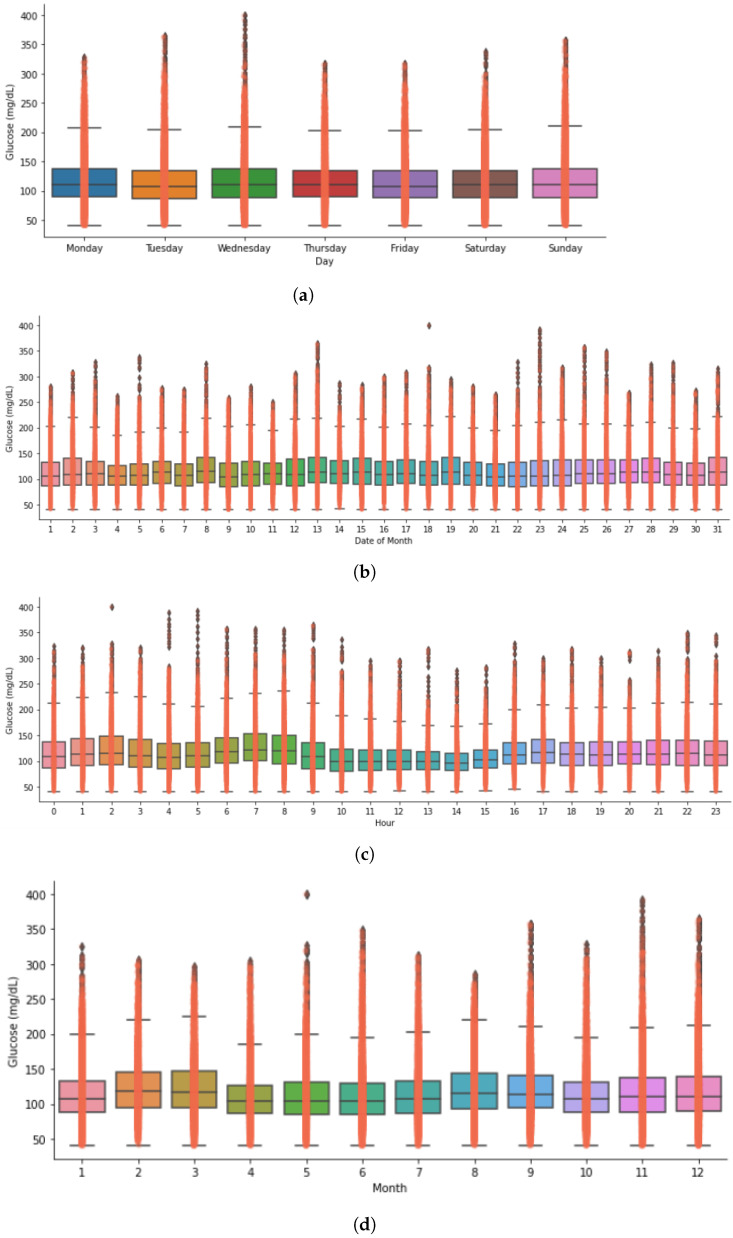
Timeseries analysis showing glucose distribution of an insulin-requiring individual using open-source AID during hours, week, days, and month. (**a**) Glucose distribution during the days of a week for an open-source AID user. (**b**) Glucose distribution during the days of a month for an open-source AID user. (**c**) Glucose distribution during the hours of a day for an open-source AID user. (**d**) Glucose distribution during the month of a year for an open-source AID user.

To perform the analysis, we separated the timestamps into hours, dates, days, and months for all the glucose entries for all the individuals and append them in separate columns as Python Pandas dataframe. We grouped the glucose entries based on hours, dates, days, and months for each individual and calculate the statistics such as count, mean, SD, minimum, maximum, Q1, Q2, and Q3. We plotted the distributions to visually inspect the differences in glucose patterns for each individual. Figure A3 shows an example for timeseries results for one insulin-requiring individual in the OpenAPS Data Commons dataset.

**Figure 6:**
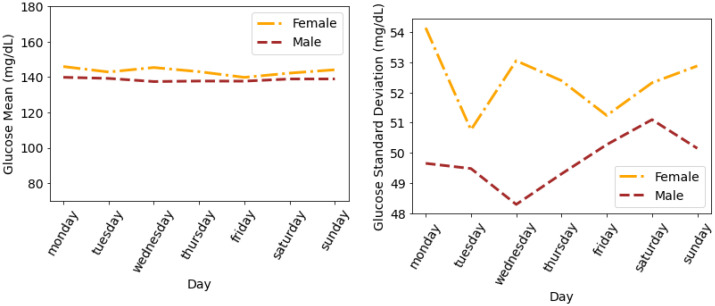
Average glucose mean and standard deviation for insulin-requiring individuals in OpenAPS Data Commons dataset during days of the week based on gender. Total number of males and females is 50 and 28, respectively.

To summarise the results and draw meaningful understandings from our dataset, we calculated and analysed the glucose mean and SD that are averaged for all individuals. Figure 6 shows the glucose mean and SD that is averaged for all individuals during the days of a week. It can be seen that both males and females follow similar trends in terms of mean glucose. The minimum mean glucose for females is 139.86 mg/dL on Friday. We also observe that the glucose mean for females is slightly greater than for males throughout the week. We observe a similar pattern in glucose SD, where females have a higher SD as compared to males; however, there is a difference in the trend of SD profiles. The maximum SD for females is 54.14 mg/dL on Monday and for males is 52.3 mg/dL on Saturday.

**Figure 7:**
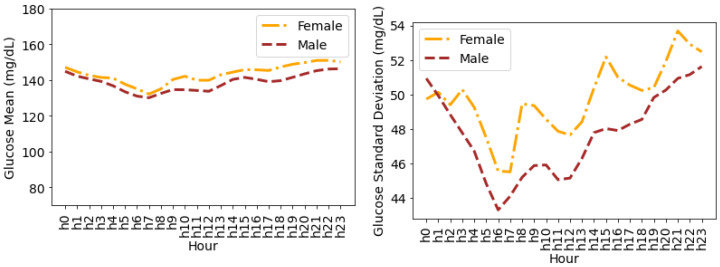
Average glucose mean and standard deviation for insulin-requiring individuals in OpenAPS Data Commons dataset during hours of a day based on gender. Total number of males and females is 50 and 28, respectively.

Figure 7 shows the glucose mean and SD that is averaged for all individuals during the hours of a day. It can be seen that the female glucose mean and SD is greater than males during all the hours of a day except for the first hour, i.e., 12:00 a.m., where male SD is greater than female SD. Both females and males have a minimum mean glucose of 145.32 mg/dL and 139.04 mg/dL at 8:00 a.m, respectively. The variations in the form of SD for both males and females drop a few hours after midnight until 8:00 a.m. and then continue to rise during the day until 12:00 a.m. The maximum SD for females and males is 49.35 mg/dL and 45.87 mg/dL during the last hour of the day (i.e., 23:00–24:00), respectively.

**Figure 8:**
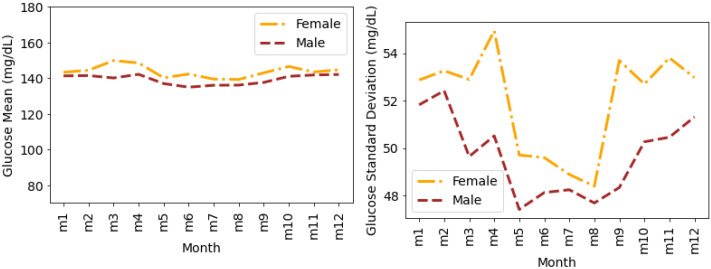
Average glucose mean and standard deviation for insulin-requiring individuals in OpenAPS Data Commons dataset during months of a year based on gender. Total number of males and females is 50 and 28, respectively. m1 represents January, m2 represents February, and similarly m12 represents December.

Figure 8 shows the glucose mean and SD that is averaged for all individuals during the months of a year. We observe a similar pattern (such as in Figure 6 and Figure 7) for glucose mean and SD with female measures greater than the males during all months of a year. It can be seen that the variations in terms of SD are greater during the first and last four months of the year. However, during the summer months (May, June, July, and August), the SD for both males and females is comparatively lower.

**Figure 9:**
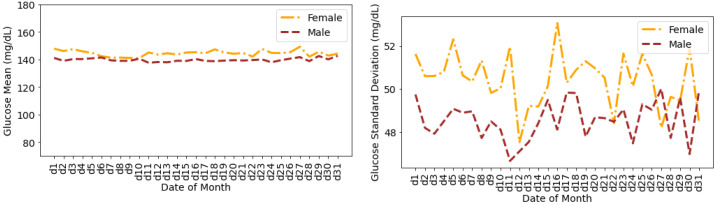
Average glucose mean and standard deviation for insulin-requiring individuals in OpenAPS Data Commons dataset during days of a month based on gender. Total number of males and females is 50 and 28, respectively.

Figure 9 shows the glucose mean and SD that are averaged for all individuals during the days of a month. The glucose mean and SD for females are greater than the males for all of the days. The variations in terms of SD are higher for females during the first 20 days of the month. There are a few overlaps between the SDs for males and females during the last 10 days of the month.

## 5. Discussion

This paper assessed demographics and glucose data analysis followed by glucose variability and timeseries analysis for diabetes-related data from the OpenAPS Data Commons, a dataset with anonymised, donated data from individuals with insulin-requiring diabetes.

Demographics were reported at the time of the first data donation, so they may not be accurate throughout the time period of glucose data available, but they nevertheless provide an opportunity to assess demographic-related correlations with glucose data. For example, within this dataset, there is a positive correlation between distributions of insulin intake and weight in females (*p* < 0.001 using Spearman correlation), whereas we observed higher carbohydrate and basal rate requirements in males. This matches existing studies showing higher carbohydrate intake in males [59] and higher weight in females with diabetes compared to males [60].

Individuals within this dataset are using open-source AID systems, which have been frequently studied and shown to achieve greater TIR [61] than recommended standards [57]. This dataset further adds to this demonstration of efficacy, with an average TIR of 77.27%. This includes data from early-era open-source AID as well as open-source AID in more recent years, which is relevant because the algorithms and feature sets of open-source AID have changed over time. As such, the TIR from this study should not only be taken as an indicator of what is currently being achieved in the “DIY” community. If such an assessment were to be preferred, a sub-analysis of the OpenAPS Data Commons could be performed looking at recent data only to reflect modern system use and a more homogeneous feature set influencing the glycemic outcomes. Additionally, early concerns from healthcare providers regarding open-source AID included the concern that open-source AID would cause additional hypoglycemia [62]; however, our analysis shows there were not excess levels of hypoglycemia alongside the overall positive efficacy observed with above-goal time in range outcomes.

Notably, within this study, machine-learning-based hierarchical clustering methods were performed, and despite the differences in glucose variability by gender described below, no obvious clusters were found. This clustering analysis was conducted to determine if there were obvious sub-populations within this dataset that were not being effectively served or were particularly effectively served by their choice of open-source AID. This is a piece of additional evidence, other than the glycemic outcomes highlighted above, in support of open-source AID being effective at solving the majority of “noise” and glucose excursions on a regular basis.

This paper is one of the first to perform an in-depth analysis of glucose variability analysis by gender. As described previously, most studies on open-source AID are simply assessing efficacy or outcomes at a population level. This study found that in this particular version of the dataset, the average LBGI for females (0.87) is lower than for males (1.11), and the average HBGI was also higher in females (4.90) than in males (4.44). However, GMI distribution patterns were not distinguished between males and females. Similarly, CV and SD within this dataset showed no distinct differences between males and females. CV distributions for both genders have negative skewness (*p* < 0.05 obtained using the Shapiro–Wilk test), whereas SD distributions are more uniform (*p* > 0.05 obtained using the Shapiro–Wilk test). Average TIR distributions between genders were also similar and uniform (*p* > 0.05), including time above range.

The timeseries analysis for glucose data based on gender provided more distinct differences between genders, although both show similar trends. The mean glucose levels for females are slightly greater than for males throughout the week, and similarly, SD is higher in females than males, although there is a different pattern throughout the week between genders. In this dataset, female SD is highest on Monday whereas males have a higher SD on Saturday (Figure 6). Moving to an assessment of the hours throughout the day (Figure 7), both females and males follow a similar pattern of glucose mean dropping between midnight and 8:00 am, then rising throughout the day and evening until midnight.

For assessing changes throughout a calendar year (Figure 8), glucose mean and SD is again higher in females than males; however, the variation in terms of SD between gender are greater in the first and last 4 months of the year. The summer months (i.e., May through August) result in lower SD for both males and females. This matches the existing population-based literature findings of seasonal variation among people with diabetes [63].

Throughout a typical calendar month, the mean glucose of females is relatively flat throughout the month but with changes in SD throughout the month. This matches findings from studies with commercial AID systems where glucose is well-managed on average by AID systems throughout the menstrual cycle [64]. However, up to two-thirds of women may experience a menstrual cycle phenomenon [65], so further analysis should be done within the female-specific sub-population to assess the two thirds who experience cyclical changes separate from those who do not. A better understanding of menstrual cycle changes [66] could lead to improvement in AID systems or education for those with menstrual cycles and their healthcare providers regarding existing features and options within AID systems that could support menstruating individuals with menstruation-related glycemic changes.

### Limitations

One of the limitations of this study is that the timeseries and gender-based analyses were performed on datasets of different lengths, e.g., some individuals may have 2–3 weeks of data while others have months and even years of data. Some analyses adjust for this, whereas others do not. These analyses are not meant to be taken as an assessment of all modern AID (open-source or commercial) but as a demonstration of methods that should be built on with additional studies.

Notably, the data within this dataset include data ranging from 2015 through 2021. Open-source AID systems have changed over time, so data from 2015 do not necessarily reflect real-world open-source AID usage in 2022 and beyond. Data from RCTs such as the CREATE trial [67] provide a modern representation of open-source AID potential instead. Lastly, the demographics are self-reported and were provided at the onset of data donation, and therefore do not necessarily match the age, weight, etc., of the individual over time.

This work primarily focused on glucose variability and demographics analysis and demonstrates the effectiveness of a large-scale data-driven analysis for the developments of open-source AID technologies. However, the OpenAPS Data Commons dataset has great potential for further applied research and development. In our future work, we will employ the data processing and analysis framework established in this paper and expand it by including algorithmic-derived novel variables such as autosenstivity [68].

## 6. Conclusions

This paper presented methods and techniques employed for anonymising, cleaning, and analysing the largest freely available diabetes dataset, i.e., OpenAPS Data Commons, donated by insulin-requiring individuals using open-source automated insulin delivery technologies. These data further validate previous findings of improved glycemic outcomes, including increased time in range without significant levels of hypoglycemia, with open-source automated insulin delivery systems. Furthermore, we employed clinically approved standard glucose variability metrics in timeseries analysis and machine-learning-based hierarchical clustering to evaluate data-driven glycemic variability outcomes. Overall, this paper showed that we can not only measure glucose variability but should be doing so within more studies. More AID studies should assess GV in addition to TIR and HbA1c and GMI, as it provides useful data for individuals living with diabetes as well as a comparison across the increasing number of AID systems becoming available. To build upon this study, future work should evaluate GV for specific situations, such as post-prandial or post-exercise, and for specific sub-populations such as menstruating individuals.

## Figures and Tables

**Table 1:** Clinically Validated Glucose Variability Metrics.

Metric	Acronym	Definition
Average daily risk range	ADRR	Assessment of overall total daily glucose variations within risk range [30]. Risk scores are defined relative to a target.
Continuous overall net glycemic action	CONGA	A GV metric similar to standard deviation (SD) that assesses glucose fluctuations for a predetermined interval [31].
Mean amplitude of glycemic excursion	MAGE	Mean of blood glucose values that exceed one SD from the 24 h mean blood glucose value [32]. Multiple implementations of automatically calculating MAGE are available in the literature [33,34,35].
Glycemic management indicator	GMI	Indicates the expected mean hemoglobin A1C using mean glucose of individuals with diabetes [36].
High blood glucose index	HBGI	A quantifying metric indicating the risk of hyperglycemia calculated using self-monitoring of blood glucose (SMBG) samples [30].
Low blood glucose index	LBGI	A quantifying metric indicating the risk of hypoglycemia calculated using SMBG samples [30].
Coefficient of variation	CV	A statistical metric to compute the diversity of glucose data. Commonly used sub-metrics for glucose data include the interday and intraday CV in CGM data [37].
Glycemic variability metric	J_index	A quality assessment metric of glucose management using a combination of information from the mean and SD [38].
Time in range	TIR	A quantifiable metric to calculate the percentage of time spent within normal glucose levels, i.e., a target range defined between 70 mg/DL to 180 mg/dL.
Time outside range	TOR	A quantifiable metric to calculate the percentage of time spent outside normal glucose levels, i.e., either less than 70 mg/DL or greater than 180 mg/dL.

**Table 2:** Count of self-reported demographics data.

Demographic Features	Number of Available Reports	Missing Reports
Total Number of Individuals	122	0
Diagnosed Date	122	0
Date of Pump Use	103	19
Date of CGM Use	105	17
Date of Closed Loop Initiation	102	20
Open-Source AID Type	107	15
Date of Birth	117	5
Country	121	1
Weight	118	4
Height	119	3
Total Daily Insulin Units	114	8
Daily Basal Insulin Units	119	3
Total Daily Carbs	105	17
Last HbA1C	116	6
Last A1C Date	116	6
Gender	78	44

## Data Availability

All programming scripts and tools developed for the analysis of demographics and glucose data in this paper are made public and online at https://github.com/danamlewis/OpenHumansDataTools/tree/master/bin/GV-demographics-scripts (accessed on 25 April 2022).

## Abbreviations

The following abbreviations are used in this manuscript:

OPEN	Outcomes of Patients’ Evidence with Novel, Do-it-Yourself Artificial Pancreas Technology
OpenAPS	Open-Source Artificial Pancreas System
AID	Automated Insulin Delivery
APS	Artificial Pancreas System
HCL	Hybrid Closed Loop
T1D	Type 1 Diabetes
CGM	Continuous Glucose Monitoring
PwD	People With Diabetes (any type)
HbA1c	Hemoglobin A1c
TIR	Time In Range
GV	Glucose Variability
